# Nuclear myosin 1 contributes to a chromatin landscape compatible with RNA polymerase II transcription activation

**DOI:** 10.1186/s12915-015-0147-z

**Published:** 2015-06-05

**Authors:** Bader Almuzzaini, Aishe A. Sarshad, Ann-Kristin Östlund Farrants, Piergiorgio Percipalle

**Affiliations:** Department of Cell and Molecular Biology, Karolinska Institute, Box 285, SE-171 77 Stockholm, Sweden; Department of Molecular Biosciences, The Wenner-Gren Institute, Stockholm University, SE-106 91 Stockholm, Sweden; Present address: National Institute of Health, National Institute of Arthritis and Musculoskeletal and Skin Diseases, Bethesda, MD 20892-3675 USA

**Keywords:** RNA polymerase II transcription, NM1, Epigenetics, Genome-wide analysis

## Abstract

**Background:**

Nuclear myosin 1c (NM1) is emerging as a regulator of transcription and chromatin organization.

**Results:**

Using chromatin immunoprecipitation and deep sequencing (ChIP-Seq) in combination with molecular analyses, we investigated the global association of NM1 with the mammalian genome. Analysis of the ChIP-Seq data demonstrates that NM1 binds across the entire mammalian genome with occupancy peaks correlating with distributions of RNA Polymerase II (Pol II) and active epigenetic marks at class II gene promoters. In mouse embryonic fibroblasts subjected to RNAi mediated NM1 gene silencing, we show that NM1 synergizes with polymerase-associated actin to maintain active Pol II at the promoter. NM1 also co-localizes with the nucleosome remodeler SNF2h at class II promoters where they assemble together with WSTF as part of the B-WICH complex. A high resolution micrococcal nuclease (MNase) assay and quantitative real time PCR shows that this mechanism is required for local chromatin remodeling. Following B-WICH assembly, NM1 mediates physical recruitment of the histone acetyl transferase PCAF and the histone methyl transferase Set1/Ash2 to maintain and preserve H3K9acetylation and H3K4trimethylation for active transcription.

**Conclusions:**

We propose a novel genome-wide mechanism where myosin synergizes with Pol II-associated actin to link the polymerase machinery with permissive chromatin for transcription activation.

**Electronic supplementary material:**

The online version of this article (doi:10.1186/s12915-015-0147-z) contains supplementary material, which is available to authorized users.

## Background

Gene expression programs are activated and repressed via ATP-dependent chromatin remodeling and epigenetic modifications. During spatial and temporal activation of genes these mechanisms target nucleosomes, DNA and histone tails [1, 2], impacting both cellular function and organismal development. By repositioning nucleosomes, ATP-dependent chromatin remodelers contribute to chromatin accessibility and exposure of DNA regulatory elements [3]. At the gene promoter, these mechanisms must be coordinated with a range of histone modifications, including acetylation, methylation, phosphorylation and ubiquitination, to collectively define different gene activity states [4, 5]. Acetylation on K9 of histone H3 (H3K9ac) by histone acetyl transferases (HAT) is commonly found at active promoters and it is therefore referred to as an epigenetic mark for active transcription. Although there are examples of enrichment at other genomic regions [6, 7], H3K4 trimethylation (H3K4me3) by histone methyl transferases (HMTs) is also associated with active chromatin, enriched at both active and poised promoters [8]. One critical histone mark that cooperates with H3K4me3 at active promoters is the modification of H3K27 by acetylation (H3K27ac) [9]. H3K27ac together with the monomethyl state of H3K4 (H3K4me1) also marks active gene enhancers [10–12]. Although both remodeling and histone modifications are essential to open up the chromatin and, thus, regulate accessibility of RNA polymerase to become engaged in active transcription, how recruitment of remodelers and active epigenetic marks is temporally orchestrated and preserved is not fully understood.

Motor proteins such as myosin are emerging as key regulators of chromatin. They coordinate global chromatin dynamics with gene-specific activities and directly affect the functional architecture of the cell nucleus [13]. Among the nuclear myosin species, the myosin 1c isoform B - referred to as nuclear myosin 1 (NM1) - is the best characterized both in terms of location and function [14–21]. NM1 works with actin and nuclear components to regulate different steps in the gene expression pathway [13, 22, 23] and has an impact at the genomic level [21]. NM1 associates with the chromatin and this association is functional since NM1 localizes to both nuclear and nucleolar transcription sites in an RNA-dependent manner [15, 19, 24–26]. At the rRNA gene promoter, the interaction between the chromatin-bound NM1 and the RNA polymerase I (Pol I)-associated actin is required for transcription activation [25]. NM1 is also part of B-WICH, a multiprotein assembly that contains the WICH chromatin remodeling complex with the subunits WSTF and the ATPase SNF2h [19, 27, 28]. On the rDNA we found that WSTF bookmarks the position of the chromatin remodeling complex while NM1 interacts with SNF2h to stabilize B-WICH, leading to recruitment of the HAT PCAF for H3K9 acetylation [25]. NM1 has therefore been proposed to connect Pol I with the rDNA through direct interactions with the Pol I-associated actin and chromatin, respectively. Since this mechanism depends on the myosin ATPase activity and the catalytic activity of NM1 is required for Pol I transcription, NM1 is likely to function as an actin-based motor that activates transcription by providing a permissive chromatin state for rapid Pol I transcription activation [20, 25, 26]. Actin also interacts with unphosphorylated RNA polymerase II (Pol II) as well as hypo- (phospho-S5) and hyperphosphorylated (phospho-S5 and phospho-S2) forms of Pol II [29–31]. There is also in vitro evidence that NM1 plays a role in Pol II transcription at different stages [13, 14, 18]. Whether the chromatin-based mechanisms described above generally apply to Pol II is, however, not known.

We set out to investigate whether NM1 has a fundamental function at class II promoters, contributing to the maintenance of a chromatin state required for Pol II transcription activation. Using chromatin immunoprecipitation and deep sequencing (ChIP-Seq), we show, for the first time, association of a myosin species with a mammalian genome. The results from pairwise comparisons of the genomic distributions of NM1 and SNF2h [3] show co-localization at multiple genomic locations. Within a subset of these locations NM1, SNF2h and WSTF are enriched at active class II promoters where NM1 specifically maintains hypophosphorylated Pol II levels and modulates B-WICH assembly, including SNF2h recruitment for local chromatin remodeling. We demonstrate that this, in turn, leads to recruitment of the HAT PCAF and the HMT Set1/Ash2, for H3K9 acetylation and H3K4 trimethylation, respectively. We propose that at class II promoters NM1 activates transcription through a chromatin-based mechanism that coordinates recruitment of chromatin remodelers and preserves active epigenetic marks.

## Results

### Measuring NM1 occupancy across the mammalian genome

**Fig. 1:**
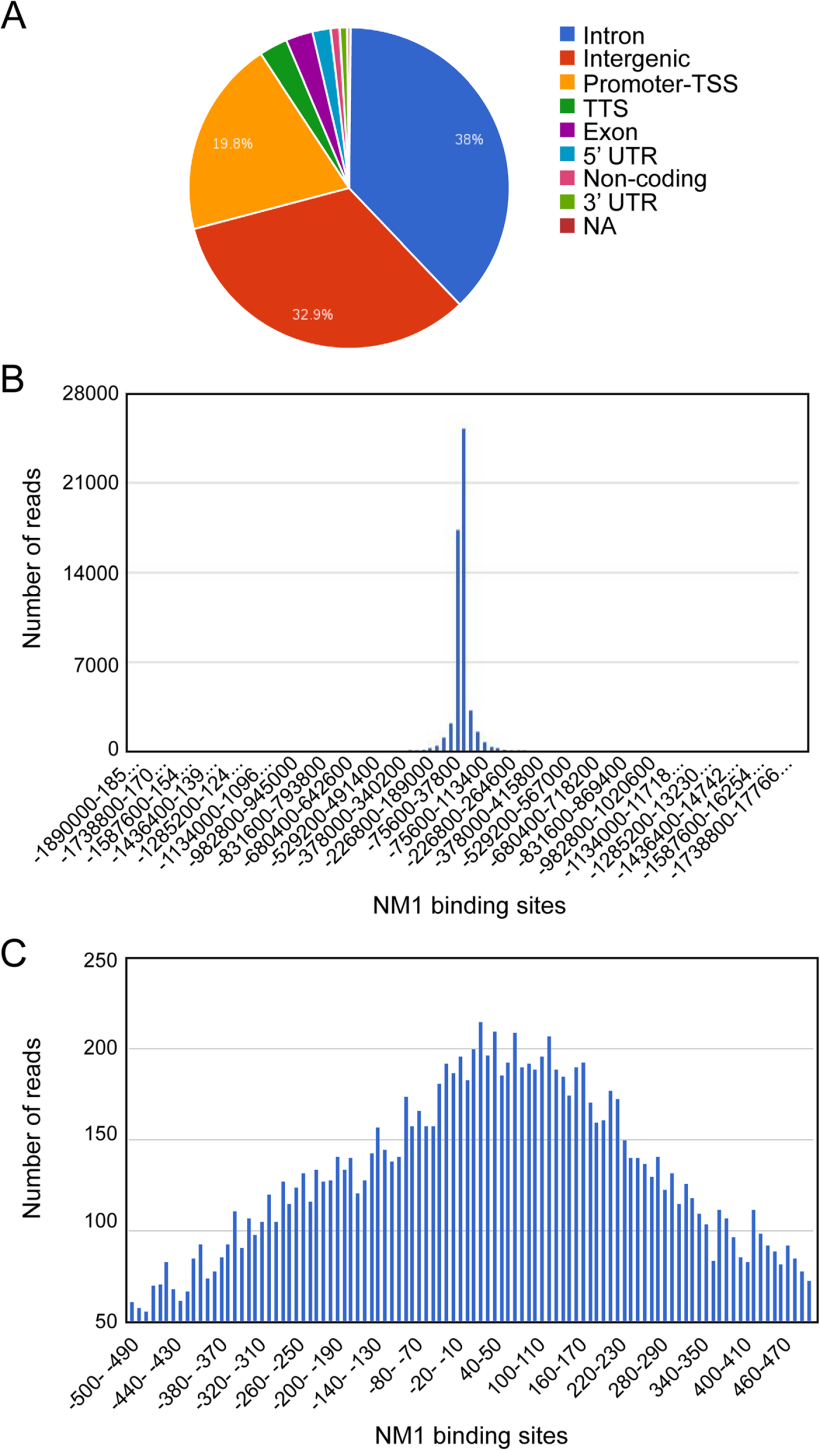
Genome wide analysis by ChIP-Seq shows that NM1 binds across the mouse genome. **a** Pie chart representation of ChIP-Seq analysis with the anti NM1 antibody showing preferred NM1 occupancies. Within genic regions NM1 is enriched at class II promoters as well as intronic and exonic sequences. **b**-**c** NM1 occupancy at class II promoters concentrates around the transcription start site (TSS), in a region comprised between −500 bp and 500 bp from TSS *bp* base pair, *ChIP-Seq* chromatin immunoprecipitation and deep sequencing, *NM1* nuclear myosin 1c

To study the distribution of NM1 across the mammalian genome, we performed chromatin immunoprecipitation and deep sequencing (ChIP-Seq) analysis using a previously characterized affinity-purified antibody specific for NM1 [15]. Briefly, after in vivo crosslinking with formaldehyde, chromatin was isolated from mouse embryonic fibroblasts (MEFs) and subjected to immunoprecipitation with the anti-NM1 antibody. The captured DNA was released by reversing the cross-linking, and the resulting DNA library was sequenced using the Illumina HiSeq 2000 platform. We identified a total of 25,738,820 reads which were then aligned against the mouse genome to map specific regions enriched for NM1. The results show that the majority of unique reads were mapped to intergenic regions, introns and promoters, including the transcription start site (TSS) and to a lesser degree, to exons and untranslated regions (Fig. 1a-c).

**Fig. 2:**
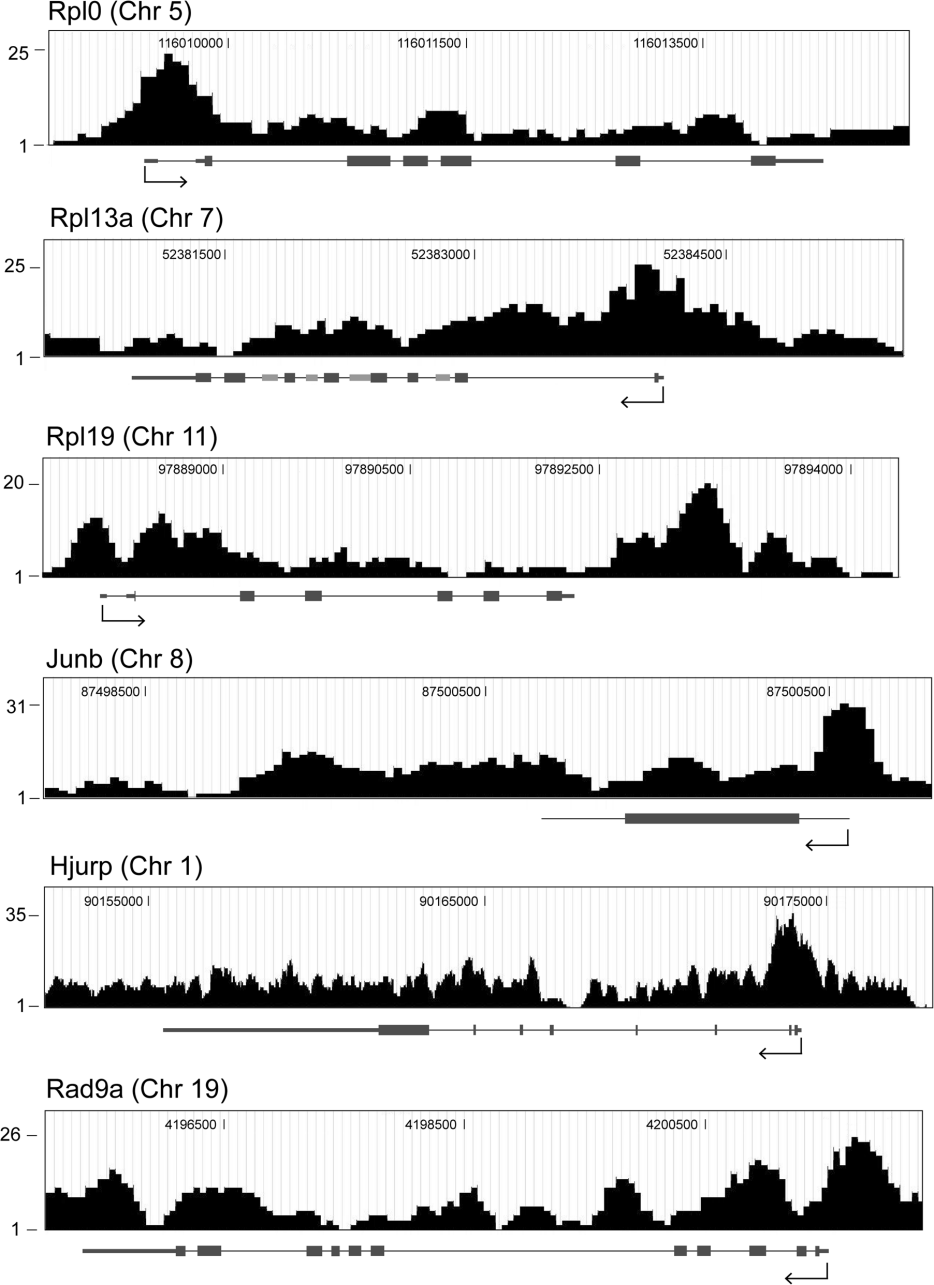
ChIP-Seq profiles showing occupancies of NM1 at selected class II genes. The profiles show peaks of NM1 occupancy at the promoters of mouse genes encoding ribosomal proteins (Rplp0, Rpl13a, RPLP19), the transcription factor Junb, Rad9a and the Holliday junction recognition protein (Hjurp) *ChIP-Seq* chromatin immunoprecipitation and deep sequencing, *NM1* nuclear myosin 1c

**Fig. 3:**
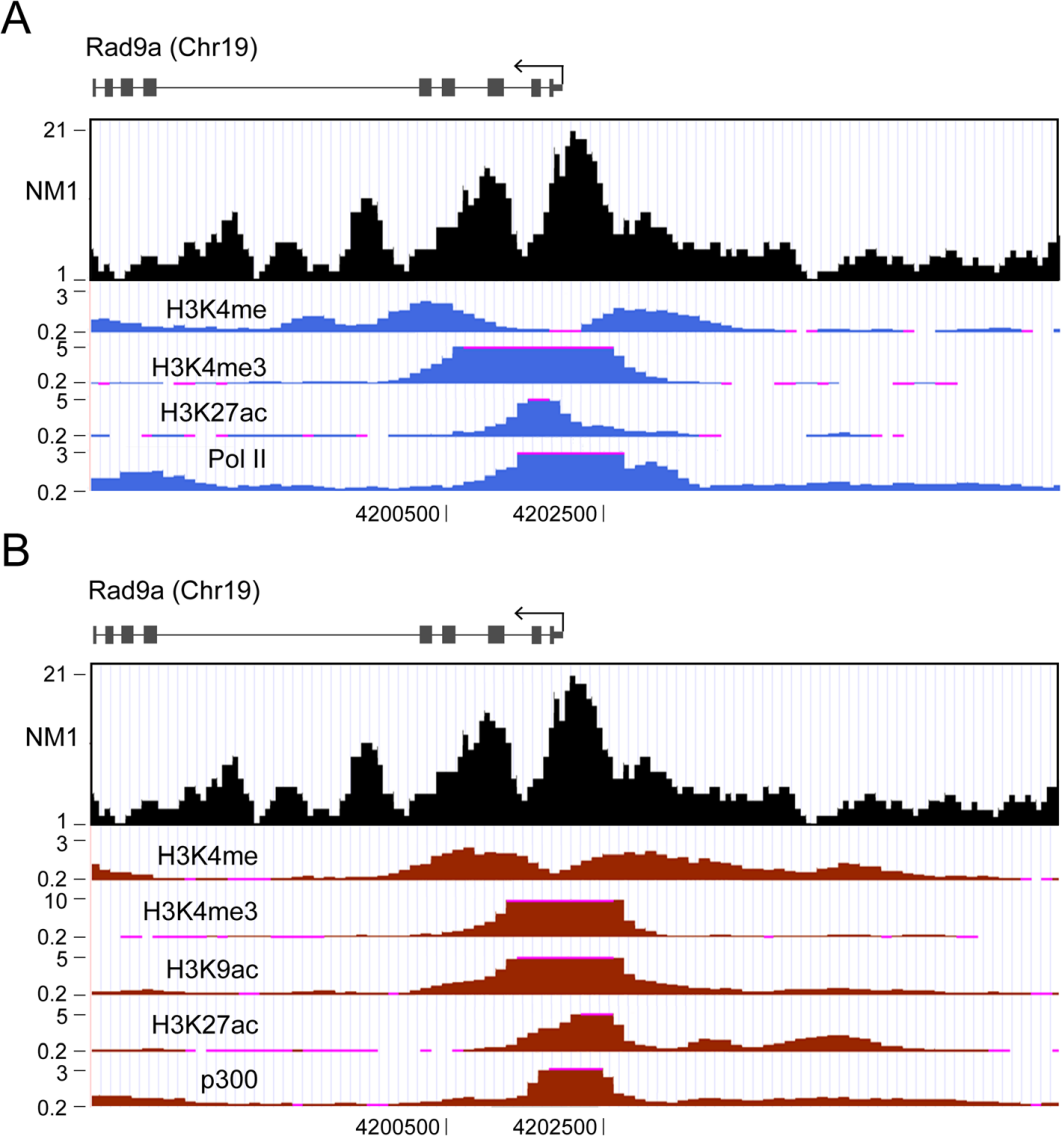
ChIP-Seq profile of the mouse Rad9a gene corresponding to the promoter region. (**a**) The ChIP-Seq profile for NM1 aligned against the mouse genome from (**a**) MEFs and (**b**) from the mouse heart correlate with the ChIP-Seq profiles for Pol II, H3K4ac, H3K27ac, H3K9ac, the HAT P300, H3K4me3 but does not correlate with H3K4me1. In the comparative analysis both MEFs and tissue (heart) were used to be able to include the H3K9ac ChIP-seq data. The tissue data used for this comparison were obtained from the UCSC browser. Importantly, in all cases the data sets were aligned against the same mouse genome (mm9) and they are therefore comparable *ChIP-Seq* chromatin immunoprecipitation and deep sequencing, *MEFs* mouse embryonic fibroblasts, *NM1* nuclear myosin 1c, *UCSC* University of California Santa Cruz

The significance of NM1 association with intergenic sequences remains to be understood. Functional clustering of peak-related genes via Gene Ontology revealed, however, that NM1 is associated with Pol II genes involved in a wide range of biological, cellular and molecular functions (Additional file 1: Figure S1; Additional file 2: Table S1). Analysis of selected ChIP-Seq profiles validated by independent ChIP/qPCR experiments showed peaks of NM1 occupancy at promoters and across the gene, at both intronic and exonic sequences (Fig. 2; Additional file 3: Figure S2; Additional file 4: Figure S3). To further characterize the localization of NM1 at the gene level, we interrogated the mouse genome to find out whether NM1 occupancy at class II promoters is compatible with the distributions of Pol II or epigenetic marks for active transcription. Using the tools available in the University of California Santa Cruz (UCSC) genome browser we analyzed several promoters such as the checkpoint clamp complex protein Rad9 gene (Rad9a), selected as NM1 binders from our ChIP-Seq analysis. We found that NM1 showed similar patterns of occupancy as Pol II, H3K4me3, H3k9ac and H3k27ac. In particular, at the gene promoter and around the transcription start site, the NM1 occupancy peaks co-localized with those of Pol II, H3K9ac, H3K27ac and H3K4me3 but did not correlate with the distribution of the epigenetic mark for active enhancers H3K4me1 (Fig. 3a and b).

It is therefore plausible that at the promoter NM1 regulates Pol II transcription and may do so through a chromatin-based mechanism that leads to correct deposition of the epigenetic marks H3K4me3, H3k9ac and H3k27ac.

### Transcription activation at class II promoters requires NM1

**Fig. 4:**
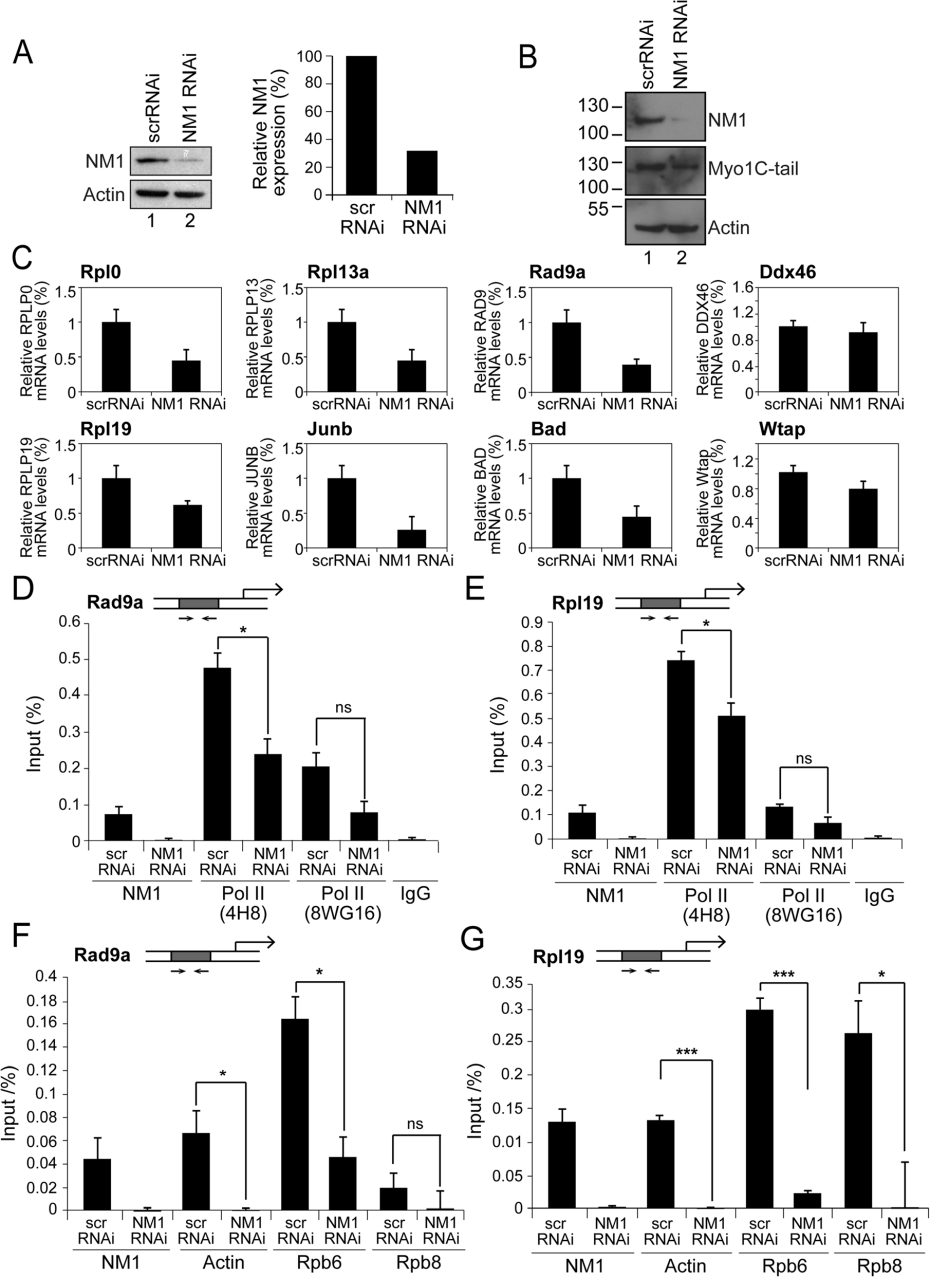
NM1 is required for the activation of Pol II transcription. **a** Steady state expression levels for NM1 and actin were analyzed on immunoblots of lysates prepared from control (scrRNAi) and NM1-silenced MEFs with an antibody to NM1 and with an antibody against actin. *Right panel*, densitometric analysis of the immunoblots for semi-quantitative measurements of NM1 steady state protein expression relative to actin. **b** Immunoblots on lysates from control (scrRNAi) and NM1-silenced MEFs with an antibody to NM1, an anti-pan-myosin 1C antibody against an epitope present in the tail of all three myosin 1C isoforms and with an antibody against actin show marginal off-target effects in the NM1 gene silencing experiment by RNAi. **c** RT-qPCR analysis of mRNA transcripts on polyA mRNA prepared from MEFs subjected to NM1 gene knockdown (NM1 RNAi) or from MEFs subjected to control siRNA oligonucleotides (scrRNAi). The mRNA levels are relative to β-actin mRNA. Error bars represent the standard deviation of three independent experiments (*n* = 3). **d-g** NM1 is required for promoter association of the active Pol II and actin. ChIP and qPCR analysis on chromatin isolated from NM1 knockdown cells (NM1 RNAi) and control cells (scrRNAi), using antibody against NM1, active (4H8) and inactive (8WG16) Pol II, actin, Rpb6 and Rpb8. In all cases, qPCR analysis was performed with primers amplifying (**d**, **f**) the mouse Rad9a gene promoter and (**e**, **g**) the mouse Rpl19 gene promoter. The values from the qPCR analyses are presented as the percentage of the input signal for each primer pair. All ChIP experiments were successfully repeated three times (*n* = 3). Error bars represent standard deviations. In panel D, p_Pol II(4H8)_ = 0.02 (*); in panel E, p_Pol II(4H8)_ = 0.015 (*); in panel F, p_actin_ = 0.05 (*), p_Rpb6_ = 0.025 (*); in panel G, p_actin_ = 0.000522 (***), p_Rpb6_ = 0.0010 (***), p_Rpb8_ = 0.01 (**). Significances were obtained by Student’s *t*-test, two-sample equal variance. ns = non-significant. In the cartoons above the bars diagrams, *arrows* point to the approximate position of primers amplifying 150 bp within the promoters *bp* base pair, *ChIP-Seq* chromatin immunoprecipitation and deep sequencing, *MEFs* mouse embryonic fibroblasts, *NM1* nuclear myosin 1c, *qPCR* quantitative polymerase chain reaction

We next evaluated whether and how NM1 may be involved in transcription regulation of class II promoters. For this, we silenced NM1 by RNAi (Fig. 4a-b). Lysates prepared from NM1-silenced cells or cells treated with control scrambled RNAi (scrRNAi) oligonucleotides were analyzed on immunoblots with anti-NM1 and anti-actin antibodies (Fig. 4a). As control against possible off-target effects in our RNAi-mediated NM1 silencing, the same membranes probed for NM1 and subsequently for actin were stripped and re-probed with an anti-pan-myosin 1C antibody against an epitope present in the tail of all three myosin 1C isoforms (Fig. 4b) (reviewed in [13]). We next isolated poly A mRNA from MEFs subjected to RNAi-mediated NM1 gene silencing and measured relative mRNA levels from several genes, selected from the NM1 ChIP-Seq results. For the analysis, we selected eight different mouse protein-coding genes covering different cellular functions. We included mouse genes encoding ribosomal proteins (Rplp0, Rpl13a, Rpl19), transcription factors (Junb), Rad9a, the pre-mRNA-splicing regulator Wtap (Wilms tumor 1-associating protein), RNA helicases (Ddx46) as well as apoptotic factors such as the BCL2-associated agonist of cell death (Bad). Results from the reverse transcription real time PCR (RT-qPCR) analysis show that NM1 gene silencing specifically induced a reduction in the relative levels of transcripts (Fig. 4c). Consistent with in vitro evidence that NM1 associates with Pol II [14], the above results suggest a possible role for NM1 in transcriptional regulation.

We next asked whether NM1 performs a potential regulatory function by facilitating Pol II association with the gene promoter. For this, we applied ChIP to crosslinked chromatin from MEFs subjected to NM1 gene silencing, using antibodies against unphosphorylated and hypophosphorylated Pol II. We also used antibodies against actin and the actin-binding core subunits Rpb6 (RPABC2) and Rpb8 (RPABC3) common to all nuclear RNA polymerases [32, 33] which are also known to directly interact with the largest Pol II subunit. The results from the qPCR analyses with primers amplifying the promoters of the mouse Rad9a and Rpl19 genes (selected from the NM1 ChIP Seq analysis among the NM1 top binders) revealed drops in the levels of hypophosphorylated Pol II, actin, Rpb6 and Rpb8 as a consequence of NM1 gene silencing whereas the levels of unphosphorylated Pol II did not significantly change (Fig. 4 d-g).

Since phosphorylation of the Pol II CTD occurs during transcription activation and marks Pol II engagement in the transcriptional process, the above results altogether suggest that NM1 may specifically activate transcription by maintaining Pol II in complex with actin at the gene promoter.

### NM1 regulates chromatin changes compatible with Pol II transcription activation

**Fig. 5:**
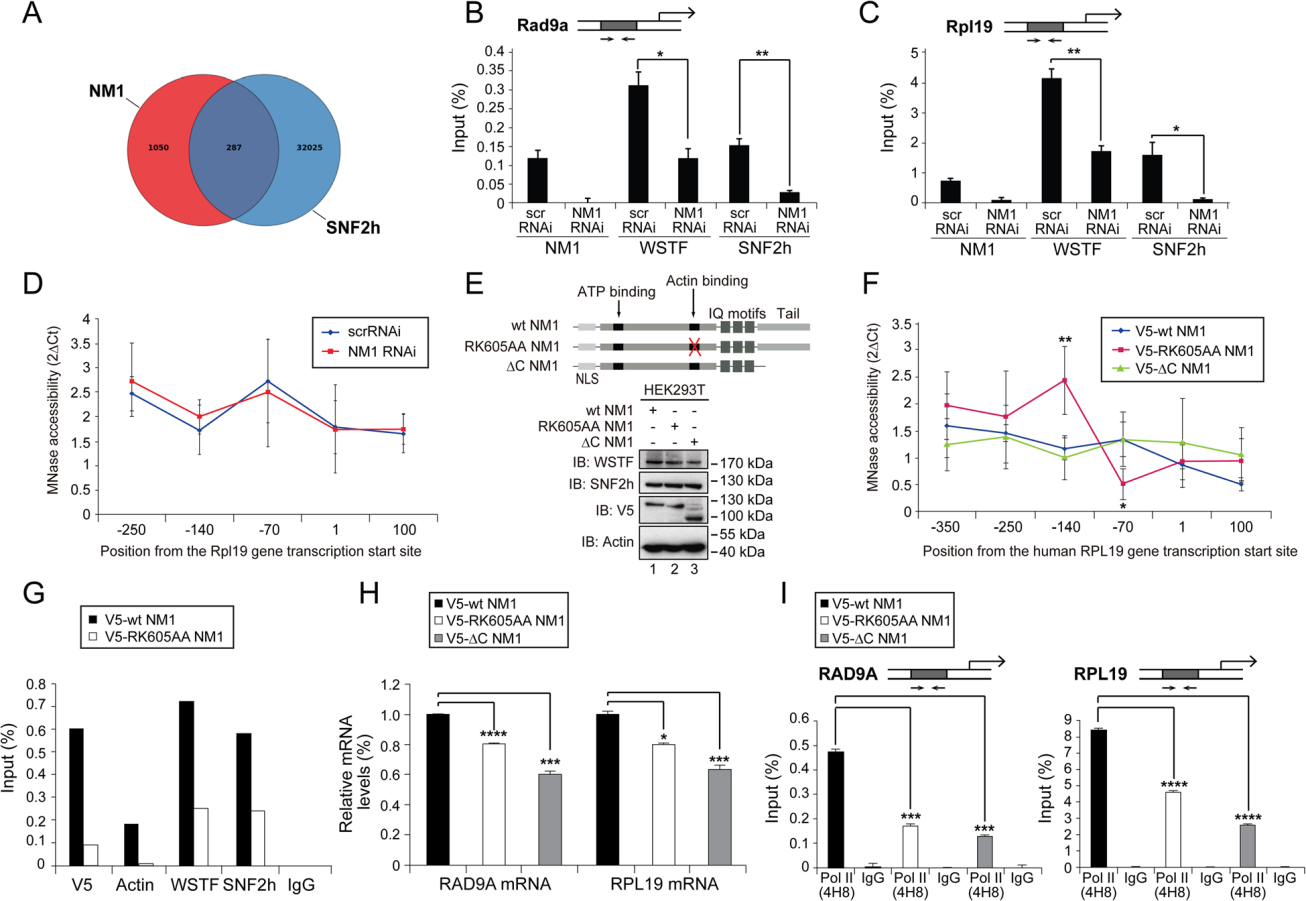
NM1 controls the levels of SNF2h at class II promoters but it is not required for chromatin remodeling. NM1 C-terminus and intact motor function are required for the association of Pol II with promoters of class II genes. **a** Venn diagrams displaying overlap of occupancy between NM1 and SNF2h across genic regions. **b**, **c** ChIP and qPCR analysis on chromatin isolated from NM1 knockdown cells (NM1 RNAi) and control cells (scrRNAi), using antibodies against WSTF, SNF2h and NM1. The qPCR analysis was performed with primers amplifying the gene promoters of (**b**) the mouse checkpoint clamp complex protein Rad9a gene (Rad9a), (**c**) the mouse ribosomal protein gene Rpl19. In all cases, the values are presented as the percentage of the input signal for each primer pair. All ChIP experiments were performed at least three time (*n* = 3). Error bars represent standard deviations. Significances (p-values) were obtained by Student’s *t*-test, two-sample equal variance. Panel B, p_WSTF_ = 0.042 (*), p_SNF2h_ = 0.0036 (**); panel C, p_WSTF_ = 0.001 (**), p_SNF2h_ = 0.017 (*). ns = non-significant. **d** Chromatin profile from HEK293T cells subjected to control (scrRNAi) or to NM1 gene silencing (NM1 RNAi) shown as 2ΔCt of undigested and MNase digested cross-linked chromatin. Similar to panel H, the position of each primer pair within the human RPL19 gene is indicated below the graph. The location +100 bp corresponds to the coding region of the gene; the location +1 bp corresponds to the transcription start site; the location −70 bp corresponds to a sequence upstream the transcription start site within the gene promoter. The results were successfully reproduced in four separate experiments (*n* = 4). Error bars represent standard deviations of four separate experiments. **e** Schematic illustration of the V5-tagged NM1 constructs expressed in HEK293T cells (top panel) and their expressions as monitored on immunoblots using anti-V5 epitope antibodies as well as expressions of actin, SNF2h and WSTF. **f** Chromatin profile from HEK293T cells stably expressing V5-wtNM1, V5-RK605AA NM1 or V5-ΔC NM1 shown as 2ΔCt of undigested and MNase digested cross-linked chromatin. The position of each primer pair within the human RPL19 gene is indicated below the graph. The location +100 bp corresponds to the coding region of the gene; the location +1 bp corresponds to the transcription start site; the location −70 bp corresponds to a sequence upstream the transcription start site within the gene promoter. The results were successfully reproduced in four separate experiments (*n* = 4). Error bars represent standard deviations of four separate experiments. An effect is detected upon expression of V5-RK605AA NM1 at −70, *p* = 0.01 (*), and at −140 *p* = 0.008 (**). **g** ChIP assays and qPCR analysis on chromatin isolated from HEK293T cells stably expressing V5-wtNM1 or V5-RK605AA NM1 using antibodies against V5, WSTF, SNF2h, actin and non-specific IgGs. qPCR analysis was performed with primers amplifying around the −70 bp region upstream the transcription start site within the human RPL19 gene promoter. The values are presented as the percentage of the input signal for each pair. The experiment was successfully reproduced two times (*n* = 2). **h** Pol II transcription analysis performed in HEK293T cells stably expressing V5-wtNM1, V5-RK605AA or V5-ΔC NM1 mutants. For the analysis, relative mRNA levels from the human genes RAD9A and RPL19 were monitored from polyA mRNA preparations by RT-qPCR using GAPDH mRNA as internal control. Error bars represent the standard deviation of three independent experiments (*n* = 3). RAD9 mRNA levels, p_RK605AA NM1_ = 5.552E-06 (****), p_ΔC NM1_ = 0.000186 (***); RPL19 mRNA levels, p_RK605AA NM1_ = 0.05543 (*), p_ΔC NM1_ = 0.000142 (***). **i** ChIP assays performed on crosslinked chromatin isolated from HEK293T cells constitutively expressing V5-wt NM1, V5-RK605AA NM1 or V5-ΔC NM1 using an antibody against the active Pol II (4H8) and non-specific IgGs. The precipitated DNA was analyzed by qPCR with primers amplifying the human RAD9A and RPL19 promoters. The bar diagrams show the relative amounts of DNA precipitated with the indicated antibodies. The values are presented as the percentage of the input signal for each pair. Error bars represent standard deviations of three independent experiments (*n* = 3). *Left panel* (RAD9 promoter occupancy), p_RK605AA NM1_ = 0.000118 (***), p_ΔC NM1_ = 0.00032 (***); *right panel* (RPL19 promoter occupancy), p_RK605AA NM1_ = 5.826E-06 (****), p_ΔC NM1_ = 5.288E-06 (****) *bp* base pair, *ChIP-Seq* chromatin immunoprecipitation and deep sequencing, *NM1* nuclear myosin 1c, *qPCR* quantitative polymerase chain reaction

We next asked whether the positive regulatory effect of NM1 on Pol II association with the gene promoter occurs through a chromatin-based mechanism. Since NM1 is part of the B-WICH complex [19], we began addressing the above question by comparing the genomic distributions of NM1 and SNF2h. A recent work mapped the different locations of SNF2h across the mouse genome [3]. Pairwise comparisons of the genomic sites occupied by NM1 and SNF2h showed that they co-localize within a considerable number of genes (Fig. 5a; Additional file 5: Table S2). Among these genes we selected some promoters and studied whether in the absence of NM1, occupancies of SNF2h as well as WSTF are altered. For this, we applied ChIP to in vivo crosslinked chromatin from NM1-silenced MEFs using antibodies to WSTF and SNF2h. The results from the qPCR analysis on the precipitated DNA show that SNF2h occupancy levels dropped across all promoters tested whereas the levels of WSTF fluctuated in a gene specific manner (Fig. 5b-c; Additional file 6: Figure S4A-D).

These results suggest that at those gene promoters where NM1 regulates association of both SNF2h and WSTF, NM1 may have an impact on the local structure of chromatin. We therefore began to look for NM1-dependent changes in chromatin accessibility by applying a high resolution micrococcal nuclease (MNase) assay [25, 34] on chromatin isolated from NM1 silenced MEFs. The results from the qPCR analyses with primers amplifying regions upstream and downstream the transcription start site (−250 kb to +100 kb) of the Rpl19 gene promoter show that knocking down the NM1 gene induced only marginal chromatin protection over the Rpl19 gene promoter at position −70 (Fig. 5d), as revealed by somewhat decreased MNase accessibility. However, the extent of chromatin protection was significantly enhanced when we performed MNase digestions on chromatin isolated from HEK293T cells stably expressing mutated NM1 constructs that function as dominant negatives in transcription [20, 25] (see also Fig. 5h). Specifically, we used HEK293T cells expressing wild-type V5-tagged NM1 (V5-wt NM1), a mutated NM1 variant that cannot bind to actin (V5-RK605AA NM1) but avidly interacts with SNF2h [25] while displaying decreased chromatin binding ability (Additional file 7: Figure S5) or a deletion construct lacking the C-terminus (V5-ΔC NM1) which cannot interact with the chromatin (Fig. 5e; Additional file 7: Figure S5; see also ref. [25]). The results from the qPCR analyses with primers amplifying regions upstream and downstream the transcription start site (−350 kb to +100 kb) of the human RPL19 gene promoter show that expression of either V5-wtNM1 or V5-ΔC NM1 did not generally affect chromatin accessibility (Fig. 5f). In contrast, stable expression of V5-RK605AA NM1 produced a significant closing of the chromatin at position −70 kb similar to the NM1 knockdown situation but considerably enhanced (Fig. 5f). The same experiment also revealed the significant establishment of a hypersensitive site further upstream at position −140 (Fig. 5f). We next performed ChIP on chromatin isolated from HEK293T cells expressing V5-wtNM1 or V5-RK605AA NM1 with antibodies to actin, WSTF and SNF2h as well as a control antibody. The results from the qPCR analysis with primers amplifying the same region around position −70 within the human RPL19 gene promoter show that expression of RK605AA NM1 induced a drop in the levels of actin, SNF2h and WSTF (Fig. 5g). These results demonstrate the requirement for a fully functional NM1 for actin association with the promoter (see also Fig. 4d and e). Moreover, these results suggest that the decreased levels of SNF2h upon stable RK605AA NM1 expression are due to SNF2h sequestration that negatively regulates chromatin, since the RK605AA NM1 mutant interacts with SNF2h and displays decreased chromatin binding efficiency. If NM1 is important for Pol II transcription activation and affects occupancies of actin and SNF2h, Pol II transcription levels should drop in the cells that stably express NM1 mutants that cannot interact with actin or with chromatin. Indeed, RT-qPCR analysis of the relative RAD9A and RPL19 mRNA levels on total polyA mRNA isolated from HEK293T cells stably expressing V5-RK605AA NM1 and V5-ΔC NM1 shows significant drops in Pol II transcription in comparison to wild-type (Fig. 5h; Additional file 7: Figure S5). Further, ChIP and qPCR analysis on chromatin isolated from HEK293T cells expressing V5-wtNM1, V5-RK605AA NM1 or V5-ΔC NM1 with antibodies to actin, hypophosphorylated Pol II CTD as well as control antibodies to non-specific IgGs show that expression of RK605AA NM1 and V5-ΔC NM1 induced drops in the levels of actin and Pol II at the promoters of the RAD9A, RPL19 genes (Fig. 5i). These findings confirm that NM1 plays a primary role in Pol II transcription and further support that NM1 activates transcription by maintaining Pol II in complex with actin at the gene promoter.

**Fig. 6:**
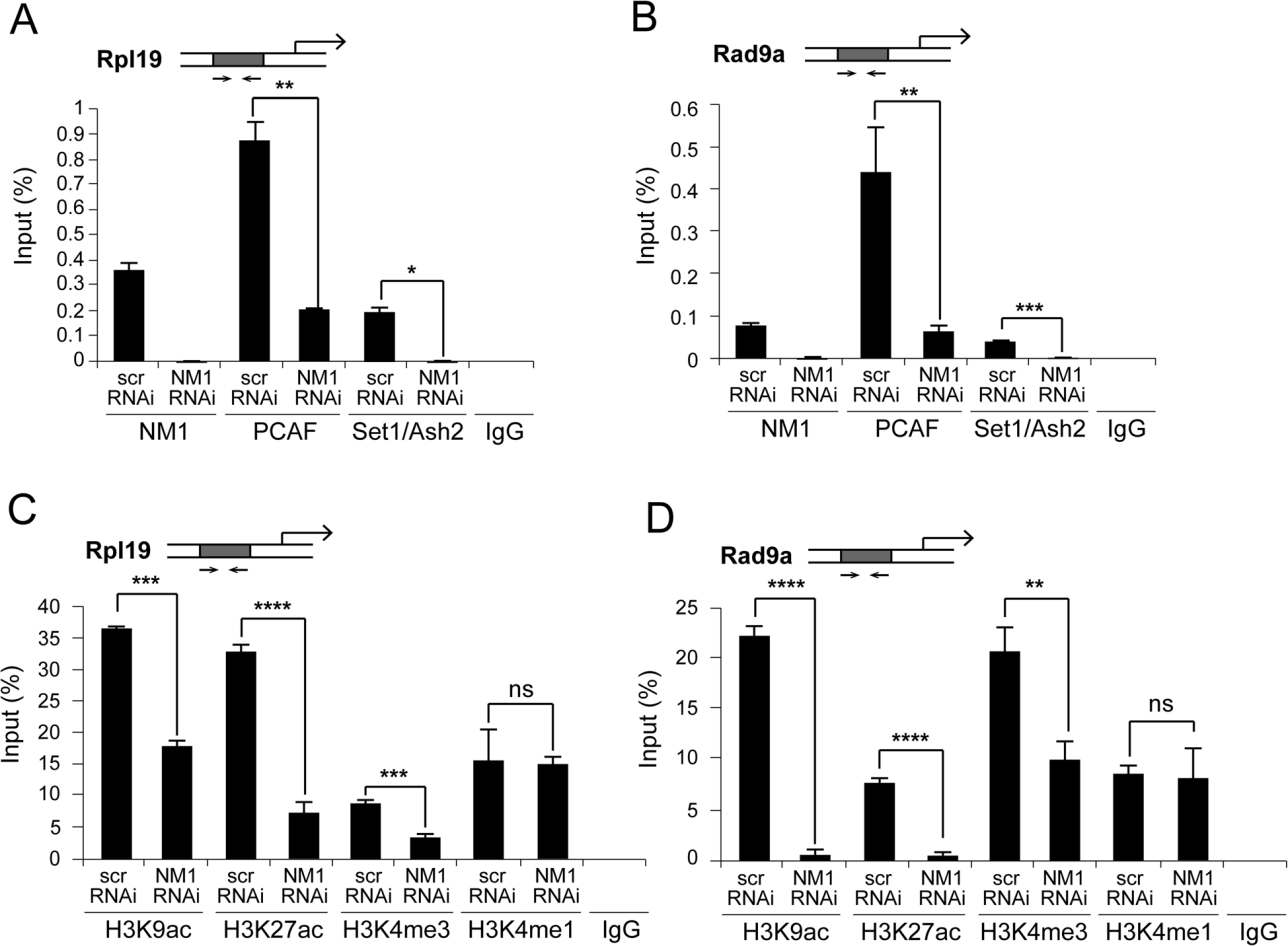
At class II promoters NM1 maintains and preserves active epigenetic marks by direct recruitment of the HAT PCAF and the HMT Set1/Ash2. **a**-**d** ChIP and qPCR analysis on chromatin isolated from NM1 knockdown MEFs (NM1 RNAi) and control MEFs (scrRNAi), using antibodies against PCAF, Set1/Ash2, H3K9Ac, H3K27Ac, H3K4me3 and H3K4me1 as well as non-specific IgGs. In all cases, qPCR analysis was performed with primers amplifying (**a**, **c**) the mouse Rpl19 gene promoter and (**b**, **d**) the mouse Rad9a gene promoter. All ChIP experiments were successfully performed at least three times (*n* = 3). The values are presented as the percentage of the input signal for each primer pair. Error bars represent standard deviations. Upon NM1 gene knockdown, significant changes were detected for PCAF, Set1/Ash2 and for the epigenetic marks H3K9Ac, H3K27ac and H3K4me3. In panel **a**, p_PCAF_ = 0.00472 (**), p_Set1/Ash2_ = 0.0214 (*); in panel **b**, p_PCAF_ = 0.01 (**), p_Set1/Ash2_ = 0.0026 (***); in panel **c**, p_H3K9ac_ = 0.000353 (***), p_H3K27ac_ = 5.93E-05 (****), p_H3K4me3_ = 0.000271 (***); in panel **d**, p_H3K9ac_ = 8.04E-05 (****), p_H3K27ac_ = 4.92E-05 (****), p_H3K4me3_ = 0.0046 (**). Significances were obtained by Student’s *t*-test, two-sample equal variance. ns = non-significant *ChIP-Seq* chromatin immunoprecipitation and deep sequencing, *HAT* histone acetyl transferase, *HMT* histone methyl transferase, *MEFs* mouse embryonic fibroblasts, *NM1* nuclear myosin 1c, *qPCR* quantitative polymerase chain reaction

We next tested whether NM1 mediates recruitment of histone modifiers. The involvement of NM1 in the regulation of H3 acetylation and methylation became plausible when we found a correlation of the NM1 peaks of occupancy with promoter-specific acetylation and trimethylation of histone H3 (Fig. 3). Furthermore, PCAF and the HMT Set1/Ash2 that respectively target H3 for acetylation and trimethylation are both part of the same complex with NM1 [25, 26]. To find out whether at class II promoters these interactions are functional, we silenced NM1 and applied ChIP using antibodies against PCAF and Set1/Ash2. The results from the qPCR analysis show that in the absence of NM1 the occupancy levels of both histone modifiers significantly dropped at all class II promoters tested, including the promoters of mouse Rpl19 and Rad9a genes (Fig. 6a and b). Importantly, at both promoters the NM1-dependent reductions in PCAF and Set1/Ash2 occupancy levels were accompanied by significant reductions in the levels of H3K9ac, H3K4me3 and H3K27ac, but not in the case of H3K4m1 (Fig. 6c and d). At class II promoters NM1 is, therefore, important for recruitment of both HATs and HMTs that will, in turn, acetylate and trimethylate H3 to generate active epigenetic marks.

We conclude that a functional NM1 is required to regulate recruitment of the chromatin remodeler SNF2h and histone modifiers in order to ensure an active chromatin state which is compatible with Pol II transcription.

## Discussion

We describe, for the first time, the functional association of a myosin motor, NM1, with a mammalian genome. ChIP-Seq analysis demonstrates that NM1 binds to both non-coding and coding regions of the mouse genome. When partitioning the coding sequences into promoter, exons, introns and UTRs, we found that NM1 is particularly enriched at class II promoters, compatible with a role for NM1 in the initial phases of Pol II transcription.

**Fig. 7:**
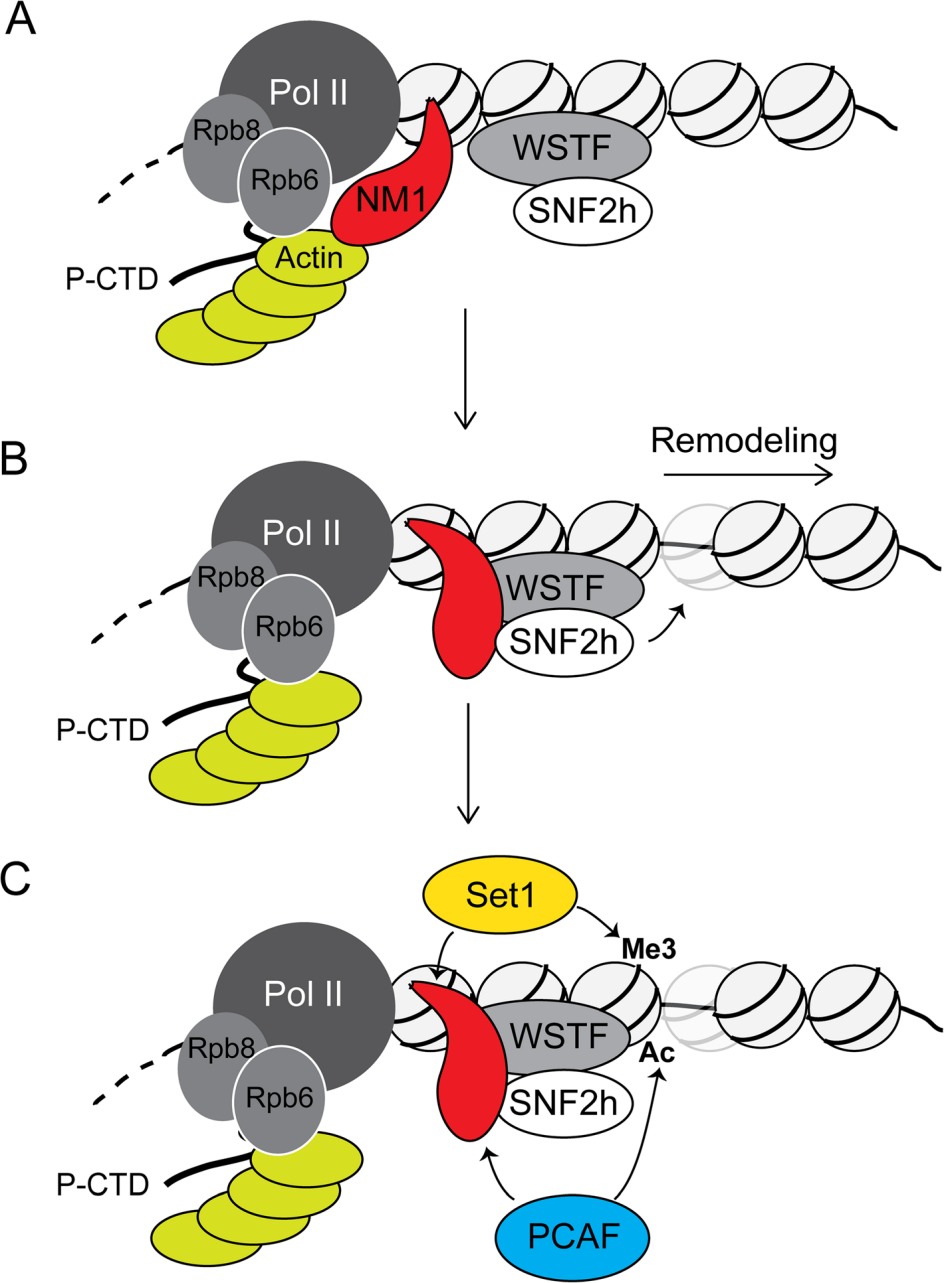
A speculative model in which NM1 coordinates the local remodeling and maintenance of active epigenetic marks for transcription activation of class II promoters. **a** NM1 interacts with actin bound to Pol II via the two core subunits Rpb6 and Rpb8. **b** NM1 can also interact with SNF2h, thus facilitating B-WICH assembly to promote remodeling. We speculate that the NM1-actin and NM1-SNF2h interactions may depend on the ATPase activity of NM1 and may exclude each other. **c** This is followed by the establishment of H3K9ac, H3K27ac and H3K4me3 through NM1-mediated recruitment of the HAT PCAF and the HMT Set1/Ash2 that ultimately leads to transcription activation *HAT* histone acetyl transferase, *HMT* histone methyl transferase, *NM1* nuclear myosin 1c

At the gene level, NM1 has been coupled to the coordination of pre-initiation complex (PIC) assembly and in transcription activation when Pol II becomes fully phosphorylated and the transcription machinery is cleared from the promoter [13]. We show evidence that NM1 gene silencing marginally affected occupancy of unphosphorylated Pol II but impaired promoter association of hypophosphorylated Pol II; this suggests that the primary function for NM1 is in transcription activation through a mechanism that facilitates loading and maintenance of the hypophosphorylated Pol II within class II promoters. We propose that NM1 performs this function in concert with actin bound to the hypophosphorylated Pol II [30, 31]. Indeed, actin itself and the two actin-binding Pol II core subunits, Rpb6 and Rpb8, efficiently interact with the promoter only in the presence of NM1. Moreover, stable expression of the RK605AA NM1 mutant, which does not efficiently interact with the chromatin and is deficient in actin-binding activity [20, 25], impairs association of actin with the gene promoter. We therefore suggest that there is a functional synergy between NM1 and actin at the gene level that is conceivably required to position the Pol II machinery at the gene promoter (Fig. 7a), possibly minimizing promoter leakage.

Our present findings also underscore the importance of the interaction between NM1 and SNF2h to start transcription at class II promoters. Genome-wide analysis by ChIP-Seq shows that NM1 and SNF2h co-localize at multiple genomic locations. The overlap between NM1 and SNF2h is not complete, consistent with a more general role of the chromatin remodeler SNF2h in the context of other nuclear functions [35]. Interestingly, however, NM1 silencing generally impaired SNF2h binding to the chromatin at class II promoters but led to gene-specific fluctuations in the levels of WSTF. These findings suggest that WSTF forms a complex with NM1 and SNF2h only at a subset of promoters. At these promoters (such as the mouse Rpl19 and Rad9a promoters), consistent with the finding that WSTF is required to co-precipitate NM1 and SNF2h from nuclear fractions [25], the WSTF-bound chromatin is likely to mark the precise location for B-WICH assembly. NM1, on the other hand, seems to play an important role in stabilizing B-WICH to exert its function. We found that stable expression of the transcriptional dominant negative RK605AA NM1 mutant led to a local closing of chromatin and a drop in the occupancy levels of SNF2h. Considering the avid interaction between the RK605AA NM1 mutant and SNF2h [25], during WSTF-dependent B-WICH assembly, NM1 possibly stabilizes the association of SNF2h with WSTF and thus with the chromatin, and this is a pre-condition for SNF2h to catalyze correct repositioning of nucleosomes (Fig. 7b). It is tempting to hypothesize that this, in turn, produces a major impact on Pol II transcription by controlling histone H1 dynamics [36]. We therefore propose that following B-WICH assembly, SNF2h-mediated remodeling opens up the chromatin to make it accessible to those histone modifications that occur immediately after repositioning of the nucleosomes and appear to be dependent on NM1. Indeed, NM1 gene knockdown led to decreased levels of epigenetic marks for active transcription, including H3K9ac, H3K4me3 and H3K27ac, but did not affect the levels of H3K4me1. These changes correlate with NM1-dependent drops of PCAF and Set1/Ash2. Therefore, it is likely that both histone modifiers are physically recruited by NM1to the gene promoter, conceivably when NM1 does not interact with actin [25]. In conclusion, we propose that the active epigenetic marks H3K9ac and H3K4me3 are under the direct control of NM1 but require a local pre-setting of the chromatin mediated by SNF2h in order to be fully executed and promote transcription activation (Fig. 7c).

## Conclusions

Class 1 myosins serve as divalent crosslinkers, physically connecting and generating force between actin filaments and cargos such as membrane lipids [37]. Our results suggest that in the cell nucleus NM1 does not seem to be an exception to the rule, although unconventional modes of action are possible. In our working model, the role of NM1 at class II promoters is dictated by its motor activity. It is linked to a cycle of attachment and detachment from dynamic Pol II-associated actin and provides a physical link between Pol II and chromatin. We speculate that the polymerase-associated actin undergoes dynamic changes in the polymerization state yet to be understood. The model translates into a temporal framework that coordinates the functions of actin in positioning hypophosphorylated Pol II at a specific site within the gene promoter, with the role of SNF2h as chromatin remodeler followed by H3K9ac, H3K4me3 and H3K27ac. This mechanism ultimately ensures that the Pol II machinery is exposed to a local chromatin landscape compatible with transcription activation as it lowers the nucleosome barrier and allows for efficient promoter clearance [38]. NM1 is enriched at the gene promoter but it also distributes across the gene. We therefore speculate that the above mechanism may also be important for Pol II transcription elongation and NM1 may synergize not only with SNF2h but with a cohort of chromatin remodelers.

Transcriptional activation is a fundamental cellular process essential for living organisms and it is spread across the entire genome. The association of NM1 with a large number of class II genes therefore argues that NM1 is a general factor involved in transcription activation. We speculate that this positive role in conjunction with actin and with the WSTF/SNF2h complex regulates genes that must be rapidly reprogrammed through chromatin de-repression in order to be activated. Nuclear actin and components of the SNF2 members of chromatin remodelers are important in transcriptional reprogramming [39–42]. A recent study has specifically shown the importance of SNF2h to establish gene expression programs underlying cerebellar morphogenesis and neural maturation in mice [36]. We therefore hypothesize that as a consequence of its motor function NM1 balls between actin and SNF2h to regulate open chromatin states, providing a genome-wide mechanism that rapidly establishes and preserves transcriptional programs during decisions important for cell fate and behavior.

## Methods

### Cell culture and reagents

Mouse embryonic fibroblasts (MEFs), wild-type HEK293T cells and HEK293T cells constitutively expressing the V5-tagged NM1 constructs V5-wtNM1, V5-RK605AA NM1 or V5-ΔC NM1 were grown in DMEM medium (Gibco, Life Technology, Carlsbad, CA, USA), supplemented with 10 % fetal bovine serum (Gibco) and a 1 % penicillin/streptomycin cocktail (Gibco) as previously performed [25, 26]. The antibodies against WSTF (ab50850), SNF2h (ab3749), H3K4m1 (ab8895), H3k4me3 (ab8580), H3K9Ac (ab10812) and H3k27ac (ab4729), Set1/Ash2 (ab70378), Rpb8 (ab104802), as well as 8WG16 (ab817) and 4H8 (ab5408) respectively targeting non-phosphorylated and hypophosphorylated (phospho-S5) heptapeptide repeats from the CTD of the largest Pol II subunit were all purchased from Abcam, Cambridge, UK. The antibodies against PCAF (sc13124) and Rpb6 (sc28711) were from Santa Cruz Biotechnology, Inc. Dallas, TX, USA. The antibodies to actin are specific for the β-isoform and were purchased from Sigma-Aldrich, St. Louis, MO, USA (clone AC74). The antibody against the V5 epitope (A190-120A) was purchased from Bethyl, Montgomery, TX, USA Laboratories. The non-specific rabbit IgGs (ab46540) were from Abcam. The antibody against NM1 has previously been characterized [15]. The anti-pan-myosin-Ic monoclonal antibody that recognizes an epitope in the tail region of myosin Ic has been previously characterized [43] and was provided by Dr. W. Hofmann (University at Buffalo-SUNY). RNAi duplexes against the target sequence GCACACGGCUUGGCACAGA in the mouse NM1 (NM1 RNAi) or control scrambled versions (scrRNAi) were purchased from Dharmacon, Lafayette, CO, USA; GE Healthcare and applied by transfection with Lipofectamine RNAi Max (Invitrogen, Waltham, Massachusetts, USA) at a final concentration of 30 nM. RNAi duplexes against human NM1 or control scrambled versions were previously described and they were applied by transfection with Lipofectamine RNAi Max (Invitrogen) at a final concentration of 30 nM as previously described [25]. The HEK293T cells stably expressing V5-wt NM1, V5-RK605AA NM1 and V5-ΔC NM1 were a kind gift of Ingrid Grummt, German Cancer Research Center, Heidelberg, Germany [20].

### Quantitative RT-qPCR

MEFs transfected with NM1 RNAi duplexes or control scrRNAi duplexes were grown in 10 cm dishes for 24 h at 37 °C. Total RNA was extracted with the TRI reagent as specified by the manufacturer’s instruction manual (Sigma). For analysis of transcripts, polyA mRNA was isolated from NM1-silenced or control cells using the Oligotex mRNA Mini Kit according to the manufacturer’s protocol (Qiagen, Venlo, Limburg, Netherlands) and treated with DNase1. cDNA was then synthesized using Superscript II reverse transcriptase (Invitrogen) using oligo dT primers according to the manufacturer’s instructions. The concentration of cDNA was determined by nanodrop. Semi-quantitative RT-qPCR was performed using the cDNA templates prepared from MEFs treated with control scrRNAi duplexes or from MEFs treated with NM1 RNAi duplexes, a Power SYBR Green PCR kit (Life Technology, Carlsbad, CA, USA), specific primers amplifying the mouse class II genes Rplp0, Rpl13a, Rpl19, Junb, Rad9a, Wtap, Ddx46 as well as Bad and the human class II genes RAD9A and RPL19 (see Additional file 8: Table S3) and a 7300 Real Time PCR System (Applied Biosystems, Waltham, Massachusetts, USA). For all primers the annealing temperature was 60 °C. All samples were run in triplicate. Relative changes in RNA levels were calculated against the reference β-actin gene using the delta-delta Ct method as previously described [25].

### Western blotting

Cells were lysed in RIPA buffer (50 mM Tris‐HCl pH 7.5, 150 mM NaCl, 1 mM EDTA, 1 % NP‐40, 0.5 % sodium deoxycholate, 0.1 % SDS) supplemented with protease inhibitors (cOmplete cocktail, Roche, Basel, Switzerland). For denaturation, protein extracts were incubated in Laemmli buffer at 95 °C for 10 min, separated by SDS–PAGE under reducing conditions and transferred to polyvinylidene difluoride (PVDF) membranes (Millipore, Billerica, Massachusetts, USA) by semidry blotting (Biorad, Hercules, CA, USA). Primary antibodies and dilutions used were NM1 (1:1000), β-actin (Sigma, 1:50000), WSTF (Abcam, 1:2000), SNF2h (Abcam, 1:500), PCAF (Abcam, 1:500), Set1/Ash2 (Abcam, 1:500). Immunoreactive bands were visualized by chemiluminescence (Amersham, GE Healthcare Life Sciences, Pittsburgh, USA).

### High-resolution MNase assay

These experiments were essentially performed as described [25, 34]. Briefly, HEK293T cells subjected to control or NM1 gene knockdown by RNAi [25], and HEK293T stably expressing V5-wtNM1, V5-RK605AA NM1 or V5-ΔC NM1 were crosslinked with 1 % formaldehyde for 20 min. Chromatin was prepared as for ChIP (see below), but washed with Buffer D containing 25 % glycerol, 5 mM magnesium acetate, 50 mM Tris (pH 8.0), 0.1 mM EDTA, 5 mM DTT. Before digestion with MNase the chromatin was lightly sonicated in MNase buffer (60 mM KCl, 15 mM NaCl, 15 mM Tris at pH 7.4, 0.5 mM DTT, 0.25 M sucrose, 1.0 mM CaCl2), 8 times for 30 s. The equivalent of 0.46 × 10^6^ cells was used in each reaction, and the level of DNA was first adjusted to be in the same range in the samples from all different treatments. Several MNase concentrations were used such that the reaction occurred in the linear range of digestion. Two samples from each treatment were used for the calculations: 10 U MNase and one concentration between 10 U and 20 U MNase. The reactions were performed at 37 °C for 30 min and then stopped by adding 12.5 mM EDTA/0.5 % SDS. After three hours of proteinase K treatment, the cross-linking was reversed at 65 °C for five hours. DNA was extracted [25] and the digest was evaluated by qPCR with primers amplifying around the transcription start site of the mouse Rpl19 gene (primers available upon request) and human RPL19 gene (for the mouse primers sequences see Additional file 9: Table S4), giving a product of approximately 100 bp. The results were analyzed by calculating ΔCt between the reactions performed with and without MNase. The values are presented as 2ΔCt. Chromatin from cells transfected with control siRNA oligonucleotides and chromatin from untransfected cells gave the same MNase digestion pattern. P-values (significances) were obtained by Student’s *t*-test as previously described [25].

### ChIP and qPCR analysis

ChIP on growing MEFs was performed as previously described [25]. Briefly, formaldehyde cross-linked chromatin was obtained from in vivo cross-linked MEFs and subjected to immunoprecipitations with antibodies to Pol II (8WG16 and 4H8), Rbp6, Rbp8, NM1, actin, WSTF, SNF2h, H3K9Ac, H3K27Ac, H3K4me1, H3K4me3, PCAF, Set1/Ash2, HDAC1 and non-specific rabbit IgGs. DNA-protein complexes were analyzed by qPCR with specific primers amplifying class II promoters (see Additional file 10: Table S5 for the primers sequences). qPCR was performed using SYBR-green from Applied Biosystems according to the manufacturer’s instructions. The primer concentration was 2.5 mM and the samples analyzed by qPCR (7300 Real Time PCR System, Applied Biosystem). The PCR conditions were: hold 50 °C for 2 min, 95 °C for 10 min, 95 °C for 15 s, 60 °C for 1 min, 95 °C for 15 s. The results were analyzed using an average of Ct of no antibody as background. The 2ΔCt of each sample in triplicate was related to the 2ΔCt of the input sample. P-values (significances) were obtained by Student’s *t*-test as previously described [25].

ChIP assays were also performed on formaldehyde crosslinked chromatin isolated from wild-type HEK293T and HEK293T cells expressing V5-wtNM1, V5-RK605AA NM1 and V5-ΔC NM1 mutants using antibodies against the V5 epitope, NM1 and histone H3 as well as non-specific rabbit IgGs. Precipitated chromatin was analyzed by PCR with primers to the EP300 gene promoter and exonic sequences and the PCR products visualized by agarose gel electrophoresis. For the primers sequences see [44].

### ChIP-Seq, sequencing, data alignment and analysis

For ChIP-Seq analysis, crosslinked chromatin from MEFs was subjected to immunoprecipitation with antibodies to NM1. A total of 5 ng of precipitated DNA was used to prepare sequencing libraries at the Bejing Genome Institute (Hong Kong) using the Illumina HiSeq 2000 platform. The analysis procedure involved the use of the SOAP2 program to map the reads to the mouse reference genome. Sequences with more than two mismatches were discarded from further analysis. The resulting individual sequences were remapped back to the annotated UCSC MM9 reference sequence which allows for the identification of peaks corresponding to the levels of association of the ChIP target with those loci. The ChIP-Seq data sets are available for download in the Gene Expression Omnibus (GEO) database (accession number GSE66542). The ChIPseek program was used to analyze the genomic distribution of NM1 across coding and non-coding elements and around the transcription start site (TSS) [45]. For this all genomic locations with a score of 10 sequences per 50 bp or above were selected. The ChIPseek program was also used for pairwise comparison of the NM1 ChIPSeq data with the SNF2h ChIPSeq data [3].

## Additional files

Additional file 1: Figure S1. Gene Onthology (GO) enrichment analysis of peak-related genes. The horizontal axis is labelled with GO items (biological processes, cellular components and molecular functions). The left vertical axis indicates the proportion of genes involved and the right vertical axis marks the exact number of genes. Additional file 2: Table S1. Lists the NM1 occupancy at Pol II genes promoters selected from the ChIP Seq data. Additional file 3: Figure S2. Selected ChIP-Seq profiles showing occupancies of NM1 at selected class II genes. The profiles show peaks of NM1 occupancy at the promoters of mouse genes encoding the proteasome (prosome, macropain) 26S subunit non-ATPase 3 (Psmd3), the RNA helicase DEAD (Asp-Glu-Ala-Asp) box polypeptide 46 (Ddx46) and the pre-mRNA-splicing regulator Wtap. Additional file 4: Figure S3. Validation of ChIP-Seq data by ChIP and qPCR analysis. For this we used selected class II promoters of mouse genes encoding the ribosomal proteins Rplp0 and Rpl19, the transcription factor (JunB), the Holliday Junction recognition protein (Hjurp), the checkpoint clamp complex protein (Rad9a), the BCL2-associated agonist of cell death Bad, the proteasome (prosome, macropain) 26S subunit non-ATPase 3 (Psmd3), the RNA helicase DEAD (Asp-Glu-Ala-Asp) box polypeptide 46 (Ddx46) and the pre-mRNA-splicing regulator Wtap. All ChIP experiments were successfully reproduced three times (*n* = 3). The values are presented as the percentage of the input signal for each primer pair. Error bars represent standard deviations. Additional file 5: Table S2. Comprises the list of genes where NM1 and SNF2h co-localize. Additional file 6: Figure S4. NM1 controls the levels of SNF2h at class II promoters. (A-C) ChIP and qPCR analysis on chromatin isolated from NM1 knockdown cells (NM1 RNAi) and control cells (scrRNAi), using antibodies against NM1, WSTF and SNF2h. The qPCR analysis was performed with primers amplifying the promoters of the mouse genes encoding (A) the transcription factor JunB gene (Junb), (B) the Holliday junction recognition protein gene (Hjurp), (C) the apoptotic factor BCL2-associated agonist of cell death gene (Bad) and (D) the ribosomal protein Rpl13a. In all cases, the values are presented as the percentage of the input signal for each primer pair. All ChIP experiments were performed at least three times (*n* = 3). Error bars represent standard deviations. Significances (p-values) were obtained by Student’s *T*-test, two-sample equal variance. Panel A, p_WSTF_ = 0.037 (*), p_SNF2h_ = 0.0042 (**); panel B, p_SNF2h_ = 0.0025 (**); panel C, p_WSTF_ = 0.0040 (**), p_SNF2h_ = 0.0017 (**); panel D, p_SNF2h_ = 0.0054 (**). ns = non-significant. Additional file 7: Figure S5. A functional NM1 is required for interaction with the chromatin. (A) Schematic representation of V5-tagged wt and mutated NM1 constructs stably expressed in HEK293T cells. (B) Schematic representation of the EP300 gene used as model gene for the ChIP assays. The numbers at the bottom of the gene structure indicate the positions of each primer used in the ChIP experiments. Steady state expressions of V5-tagged wt and mutated NM1 constructs were monitored on immunoblots for V5 and actin antibodies as loading control. (C) ChIP analysis on chromatin isolated from wild type HEK293T cells or HEK293T cells expressing V5-wtNM1, V5 RK609AA NM1, V5-ΔC NM1 and V5-ΔIQ NM1, using an antibody against the V5 epitope, antibodies against histone H3 and non-specific rabbit IgGs. In all cases, the ChIP was analyzed by PCR, agarose gel electrophoresis and ethidium bromide staining. Bottom parts, densitometric quantification of relative occupancies. Bar diagrams represent values calculated from analysis of exon 3, exon 6 and exon 31 in duplicates. All values are normalized against the signals obtained with the anti-V5 antibody targeting the V5-wt NM1 construct. Additional file 8: Table S3. Listing primers used in the RTqPCR analysis of mRNA levels upon NM1 gene knockdown. Additional file 9: Table S4. Listing primers targeting the human RPLP19 gene promoter, used in the MNase/qPCR assay. Additional file 10: Table S5. Listing primers used in the ChIP/qPCR analyses.
