# Supplementary figures and images for: Nuclear myosin 1 contributes to a chromatin landscape compatible with RNA polymerase II transcription activation
Source: BMC Biol. 2015 Jun 5;13:35. doi: 10.1186/s12915-015-0147-z (PMC4486089; doi:10.1186/s12915-015-0147-z)

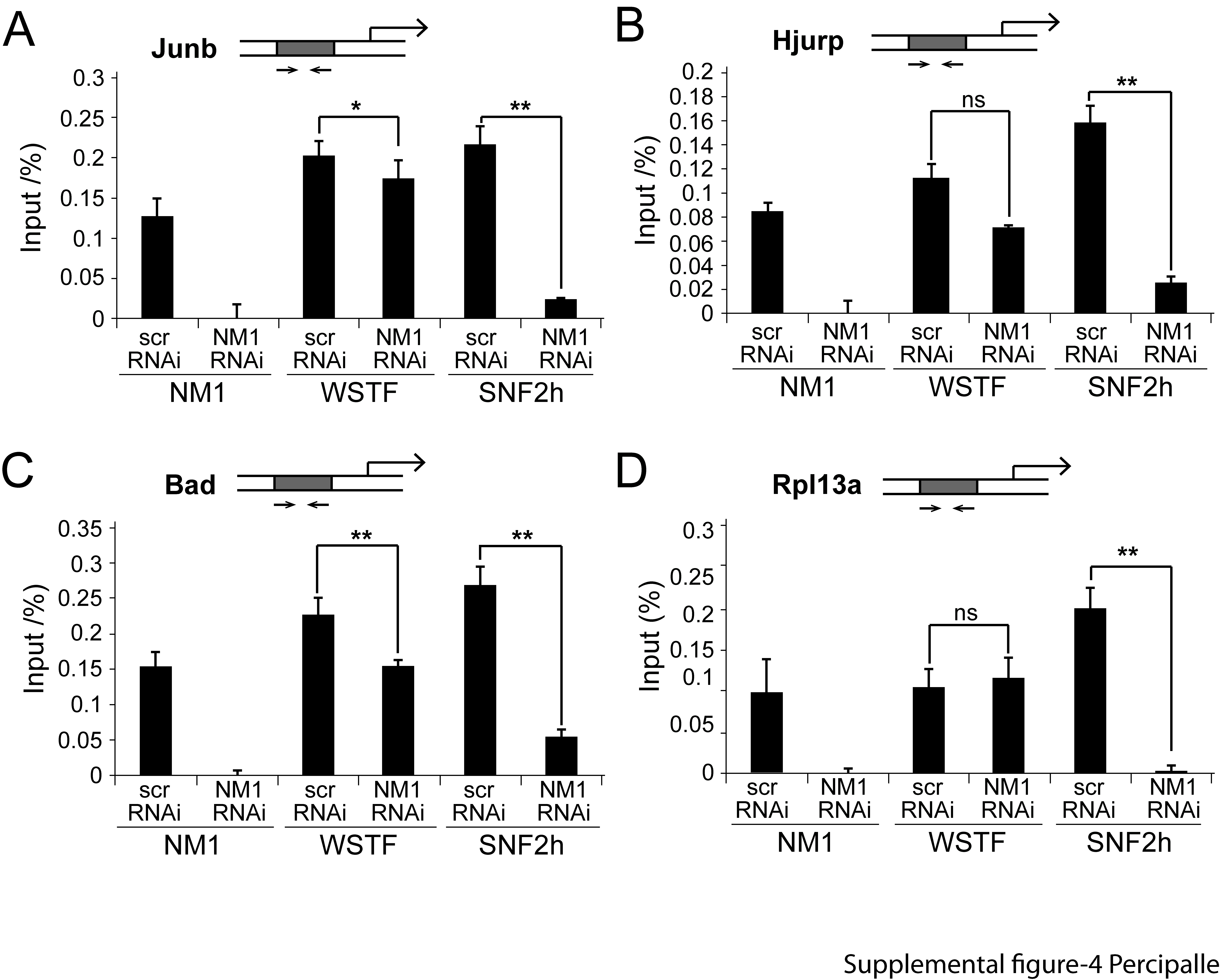

Supplement: Additional file 6: Figure S4. — NM1 controls the levels of SNF2h at class II promoters. (A-C) ChIP and qPCR analysis on chromatin isolated from NM1 knockdown cells (NM1 RNAi) and control cells (scrRNAi), using antibodies against NM1, WSTF and SNF2h. The qPCR analysis was performed with primers amplifying the promoters of the mouse genes encoding (A) the transcription factor JunB gene (Junb), (B) the Holliday junction recognition protein gene (Hjurp), (C) the apoptotic factor BCL2-associated agonist of cell death gene (Bad) and (D) the ribosomal protein Rpl13a. In all cases, the values are presented as the percentage of the input signal for each primer pair. All ChIP experiments were performed at least three times (n = 3). Error bars represent standard deviations. Significances (p-values) were obtained by Student’s T-test, two-sample equal variance. Panel A, pWSTF = 0.037 (*), pSNF2h = 0.0042 (**); panel B, pSNF2h = 0.0025 (**); panel C, pWSTF = 0.0040 (**), pSNF2h = 0.0017 (**); panel D, pSNF2h = 0.0054 (**). ns = non-significant. [file 12915_2015_147_MOESM6_ESM.tiff]
