# Supplementary material for: Nuclear myosin 1 contributes to a chromatin landscape compatible with RNA polymerase II transcription activation
Source: BMC Biol. 2015 Jun 5;13:35. doi: 10.1186/s12915-015-0147-z (PMC4486089; doi:10.1186/s12915-015-0147-z)
Supplement: Additional file 8: Table S3. — Listing primers used in the RTqPCR analysis of mRNA levels upon NM1 gene knockdown. [file 12915_2015_147_MOESM8_ESM.doc]

| **Reverse** | **Forward** | **Gene** |
| --- | --- | --- |
| 5’ TCGGGTCCTAGACCAGTGTTC | 5’ AGATTCGGGATATGCTGTTGGC | Rplp0 (mouse) |
| 5’ GCTTCTTCTTCCGATAGTGCATC | 5’ AGCCTACCAGAAAGTTTGCTTAC | Rpl13a (mouse) |
| 5’ GCATTGGCGATTTCATTGGTC | 5’ ATGAGTATGCTCAGGCTACAGA | Rpl19 (mouse) |
| 5’ CCTTGAGACCCCGATAGGGA | 5’ TCACGACGACTCTTACGCAG | Junb (mouse) |
| 5’ GCTCACTCGGCTCAAACTCT | 5’ AAGTCCGATCCCGGAATCC | Bad (mouse) |
| 5’ GTGGGGCAAAAAGGAAGCAG | 5’ GGCTGTCCATTCGCTATCCC | Rad9a (mouse) |
| 5’ AGCTATTGGTCCCTCTCCTTC | 5’ ATGTCTGGACGAGATTTGATTGG | Ddx46 (mouse) |
| 5’ TTAACTCATCCCGTGCCATAAC | 5’ GAACCTCTTCCTAAAAAGGTCCG | Wtap (mouse) |
| 5' GCC AGG TGA AAG GGA AAT GG | 5’ CAT TGA CTC TTA CAT GAT CGC CA | RAD9A (human) |
| 5’ TCA GGT ACA GGC TGT GAT ACA | 5’ GGG CAT AGG TAA GCG GAA GG | RPL19 (human) |

**Supplemental table 3**. List of primers used in the RTqPCR analysis of mRNA levels in mouse embryonic fibroblasts and in HEK293T cells.
