# Supplementary material for: Nuclear myosin 1 contributes to a chromatin landscape compatible with RNA polymerase II transcription activation
Source: BMC Biol. 2015 Jun 5;13:35. doi: 10.1186/s12915-015-0147-z (PMC4486089; doi:10.1186/s12915-015-0147-z)
Supplement: Additional file 9: Table S4. — Listing primers targeting the human RPLP19 gene promoter, used in the MNase/qPCR assay. [file 12915_2015_147_MOESM9_ESM.doc]

**Supplemental table 4.** List of primers targeting the human RPL19 gene promoter, used in the MNase/qPCR assay.

| **Primer name** | **Primer sequences** |
| --- | --- |
| -350 F | 5’ CATCCACCCACAACCCCCC |
| -350 R | 5’ GTAACCGGAAGTCAGCTGG |
| -250 F | 5’ CCAGCTGACTTCCGGTTAC |
| -250 R | 5’ CCCCTTCCGTAGGGAAAGT |
| -140 F | 5’ ACTTTCCCTACGGAAGGGG |
| -140 R | 5’ GTTCTTTCATCTTCTGCCACAC |
| -70 F | 5’ GTGTGGCAGAAGATGAAAGAAC |
| -70 R | 5’ CTCCTCCCATTATCTGCGAAG |
| +1 F | 5’ CTTC**GCA**GATAATGGGAGGAG |
| +1 R | 5’ GTCAGCGCCTGATGGAGAC |
| +100 F | 5’ GTCTCCATCAGGCGCTGAC |
| +100 R | 5’ GAGGCCCGGGACGAAAGTG |
|  |  |
