# Supplementary material for: Nuclear myosin 1 contributes to a chromatin landscape compatible with RNA polymerase II transcription activation
Source: BMC Biol. 2015 Jun 5;13:35. doi: 10.1186/s12915-015-0147-z (PMC4486089; doi:10.1186/s12915-015-0147-z)
Supplement: Additional file 10: Table S5. — Listing primers used in the ChIP/qPCR analyses. [file 12915_2015_147_MOESM10_ESM.doc]

**Supplemental table 5**. List of primers targeting mouse and human promoters used in the ChIP/qPCR analyses.

| **Reverse** | **Forward** | **Gene** |
| --- | --- | --- |
| 5’ CCCACAACAAAACAACCCCC | 5’ CCTATAAAAGGCACACGCGG | Rplp0 (mouse) |
| 5’ AAACCCTGCGACAAGACCTC | 5’ TGCTAAGTCCCATCGCACAA | Rpl13a (mouse) |
| 5’ AGTACAGGCTGGAGATGGCT | 5’ TACACCAGAAGAGGGCATCC | Rpl19 (mouse) |
| 5’ CCGCAGCTGACAAATTCAGT | 5’ CGGAGATTTTCTCTCCCTCCG | Junb (mouse) |
| 5’ CATGGAGTCTATGGGTCGGC | 5’ TCGCTATCAGCCGCTTCATT | Hjurp (mouse) |
| 5’ ATTGCGGAACCCCAAACGAA | 5’ GTCGCGAGAAACGTGCTTTA | Bad (mouse) |
| 5’ CCACACCCAATGAACCGACT | 5’ TATTGGTTCACCCGGGGTTT | Rad9a (mouse) |
| 5’ CGGTTCTTGTTCCCCTCCCG | 5’ CTTCGGTCCCCGGACTAGG | Psmd3 (mouse) |
| 5’ CAGCCCTTTTTGGCGCTATG | 5’ AGGCTGCATTAGGTTCCTCG | Ddx46 (mouse) |
| 5’ TACTCCAGAACCCTCACCCG | 5’ AAGCGCCCCTCGTTTTAAGT | Wtap (mouse) |
| 5’ GGC AGA GGG AAG AAC AAG AG | 5’ CAA AAA AAG GCA CAT AAC CAC G | RAD9A (human) |
| 5’ TCA TCT TCT GCC ACA CTA AAC | 5’ AAA ACA TCC ACC CAC AAC C | RPL19 (human) |
